# Laypersons versus experienced surgeons in assessing simulated robot-assisted radical prostatectomy

**DOI:** 10.1007/s00345-023-04664-w

**Published:** 2023-10-26

**Authors:** Rikke Groth Olsen, Lars Konge, Khalilullah Hayatzaki, Mike Allan Mortensen, Sarah Hjartbro Bube, Andreas Røder, Nessn Azawi, Flemming Bjerrum

**Affiliations:** 1 Copenhagen Academy for Medical Education and Simulation (CAMES), University Hospital - Rigshospitalet, Ryesgade 53B, 2100 Copenhagen, Denmark; 2 Department of Urology, Copenhagen Prostate Cancer Center, Copenhagen University Hospital - Rigshospitalet, Copenhagen, Denmark; 3 Faculty of Health and Medical Sciences, University of Copenhagen, Copenhagen, Denmark; 4 Department of Urology, Zealand University Hospital, Roskilde, Denmark; 5 Department of Urology, Odense University Hospital, Odense, Denmark; 6 Department of Clinical Research, University of Southern Denmark, Odense, Denmark; 7 Department of Surgery, Herlev-Gentofte Hospital, Herlev, Denmark

**Keywords:** Crowdsourcing, Urology, Prostatectomy, Robotic surgical procedures, Assessment, Surgical education

## Abstract

**Background:**

Feedback is important for surgical trainees but it can be biased and time-consuming. We examined crowd-sourced assessment as an alternative to experienced surgeons’ assessment of robot-assisted radical prostatectomy (RARP).

**Methods:**

We used video recordings (*n* = 45) of three RARP modules on the RobotiX, Simbionix simulator from a previous study in a blinded comparative assessment study. A group of crowd workers (CWs) and two experienced RARP surgeons (ESs) evaluated all videos with the modified Global Evaluative Assessment of Robotic Surgery (mGEARS).

**Results:**

One hundred forty-nine CWs performed 1490 video ratings. Internal consistency reliability was high (0.94). Inter-rater reliability and test–retest reliability were low for CWs (0.29 and 0.39) and moderate for ESs (0.61 and 0.68). In an Analysis of Variance (ANOVA) test, CWs could not discriminate between the skill level of the surgeons (*p* = 0.03–0.89), whereas ES could *(p* = 0.034).

**Conclusion:**

We found very low agreement between the assessments of CWs and ESs when they assessed robot-assisted radical prostatectomies. As opposed to ESs, CWs could not discriminate between surgical experience using the mGEARS ratings or when asked if they wanted the surgeons to perform their robotic surgery.

## Introduction

Most surgical errors occur during the beginning of a surgeon’s learning curve [1]. Therefore, effective training and assessment are essential to ensure patient safety by ensuring surgeons possess the necessary competencies [2]. Traditionally, surgical assessment is performed by direct observations by an expert; however, direct assessments are subject to several biases. The expert could stick to a previous opinion of the trainee and assess based on personal feelings rather than the actual performance [3, 4]. Experts are a limited resource so new methods of assessment need to be more feasible and scalable [5, 6].

In a modern healthcare system, we rely more than ever on using patient knowledge to guide clinicians in everyday encounters with patients. “Patient-focused medicine” has given the patient a more central role [7] and WHO has set patient empowerment as a key element for improving healthcare outcomes [8]. Through crowdsourced assessment, patients can contribute to medical education and research from a layperson’s perspective with their healthcare experiences [7].

Crowd-sourced assessment is a process that utilizes laypersons to complete online tasks either paid or unpaid [9]. The knowledge of the crowd workers (CWs) is used to assess a surgeon’s technical skills, e.g., robotic suturing without specific training in surgical skills [5, 6]. Through internet-based ratings, a single video can get several hundred ratings by CWs in a couple of hours, which can rapidly help the surgeon identify what she/he could gain from further training [6, 10].

In a comparative, blinded assessment study, we examined the use of laypersons as crowd workers. We compared the assessment of the laypersons with those of experienced surgeons for assessing the performance of robot-assisted radical prostatectomy (RARP). Further, we explored if some types of CWs are better at assessment than others.

## Materials and methods

### Participants

The CWs were recruited by a Danish Association for volunteer, unpaid patients who want to contribute to research, *Forskningspanelet *(Copenhagen, Denmark, https://forskningspanelet.dk/). All CWs were inexperienced with video rating and assessment of RARP. We aimed to recruit a minimum of 68 CWs to receive ratings similar to ESs’ ratings [11]. All CWs filled out a demographic questionnaire and informed consent at the start of the project.

Two experienced RARP surgeons (ESs) were invited as expert raters (110–150 RARP procedures performed).

### Video material

We used video recordings of surgeons on a robotic VR simulator, the RobotiX Mentor™ (Surgical Science^®^, Sweden) as in a previously published study [12, 13]. We randomly chose videos from five novice surgeons (novices), five experienced robotic surgeons (intermediates), and five experienced robotic surgeons in RARP (experienced). The novices were residents in urology who had assisted to a minimum of one RARP but had not performed any robot-assisted surgeries. The intermediates were experienced robotic surgeons in urology but had never performed RARPs. The experienced RARP surgeons primarily performed RARP during their clinical work and seldom other urological robot-assisted surgeries.

We used video recordings on three different modules: bladder neck dissection (BND), neurovascular bundle dissection (NVBD), and urethrovesical anastomosis (UVA), we edited videos to include the first 5 min to standardize them. We anticipated that the total time for video assessment for the CWs would be too long [14] as full-length videos were up to 43 min long. All CWs and ESs were blinded to the identity and skill level of the surgeon on the recorded video.

### Assessment tool

CWs and ESs rated the videos using the assessment tool, modified Global Evaluative Assessment of Robotic Skills (mGEARS), which comprises five domains: depth perception, bimanual dexterity, efficiency, force sensitivity, and robotic control. Performance in each domain is measured on a five-point Likert scale. A rating of 1 corresponded to the lowest level of performance and a rating of 5 corresponded to the highest level of performance. An overall performance rating is derived by summing the ratings of each of the domains (5–25 points) [15].

### Rater training and assessment

An elaborate explanation of the assessment tool, mGEARS, was given to the CWs including how to rate each video. They were given a brief explanation, including an illustration of the anatomy and the purpose of the part procedures. Each CW rated ten randomly distributed videos. After rating each video, the CWs were asked to answer ‘Yes/No’ to the question: ‘Would you trust this doctor to perform robot-assisted surgery on you?’. In the end, the CWs received a final questionnaire regarding time use and possible payment level.

The ESs were rater trained to expertise to ensure they represented the ‘gold standard’. The ESs were presented with the same explanation of the assessment tool, introduction of anatomy, and purpose of the part procedures as the CWs. ESs assessed six videos with mGEARS from each part of the surgery from novice surgeons and experienced surgeons. Their ratings were compared and discussed until an agreement on assessment level was reached. These videos were not included for assessment in the study. The ES then rated all 45 videos blinded and independently.

### Ethics

Approval by The Danish Data Protection Agency was secured before enrollment (P-2020-701) and the study was deemed exempt from ethical approval by The Danish National Ethics Committee (H-20023590). All videos were pseudo-anonymized with a randomly allocated identification ID and all participants received a unique link only known to the participant and the principal investigator (RGO).

### Statistical analysis

We examined the internal consistency reliability test (across mGEARS items) for the CWs and ESs to test if we could use total mGEARS ratings for each part of the procedure. We calculated inter-rater reliability tests and test–retest reliability for both CWs and ESs to test the use of total performance ratings for each surgeon performing the simulated RARP. We performed Analysis of Variance (ANOVA) and intergroup comparisons using independent samples *t* tests to test if CWs and ESs could discriminate between groups with different surgical experiences (novices, intermediates, and experienced). The statistical significance level was set at 0.05 [16].

Furthermore, using a delta mean score, we tested how accurately each CWs rated the videos compared to the ESs. We calculated the delta mean score by the difference between the mean GEARS ratings of the two ESs for each of the 45 videos and the rating each CW gave the videos giving us a total of 1490 delta mean scores. A delta score of zero would mean a total agreement between the CW and ESs. We then performed a Pearson’s correlation test or an independent *t* test to see if any type of CW performed better than other CWs based on age, gender, health care education, and their answers to the final questionnaire.

We used an independent *t* test calculated between the mean delta scores of the CWs to test if the CWs’ opinion about a future role in crowd-sourced assessment influenced their performance.

Finally, a Chi-square test was used to analyze the CWs’ answer to the question: ‘Would you trust this doctor to perform robot-assisted surgery on you?’.

## Results

One hundred forty-nine CWs performed a total of 1490 video ratings (22–45 ratings per video). The two experienced RARP surgeons rated all 45 videos with a total of 90 video ratings (2 ratings per video).

Inter-rater reliability (0.29) and test–retest reliability (0.39) for CWs were low. As a result of the low test–retest reliability of the CWs, we performed an ANOVA and independent *t* test across ratings of part procedures and not total performance scores. As shown in Table 1, the CWs could discriminate between novice and experienced surgeons performing NVBD (*p* = 0.03) but all other comparisons were not significant (*p* = 0.10–0.89).

**Table 1:** CWs’ mGEARS ratings for novices, intermediates, and experienced surgeons for the three part procedures using ANOVA (Analysis of Variance) and independent *t* test

	BND	NVBD	UVA
Novice, mean (SD)	17.44 (1.26)	14.10 (0.75)	15.97 (3.92)
Intermediate, mean (SD)	16.48 (2.69)	14.90 (2.46)	16.95 (2.20)
Experienced, mean (SD)	18.19 (1.54)	17.02 (2.38)	16.47 (3.44)
Between groups,* p*	0.40	0.10	0.89
Novice surgeons versus intermediate surgeons,* p*	0.49	0.52	0.64
Intermediate surgeons versus experienced surgeons,* p*	0.25	0.21	0.80
Novice surgeons versus experienced surgeons,* p*	0.43	0.03	0.84

*BND* bladder neck dissection, *NVBD* neurovascular bundle dissection, *UVA* urethrovesical anastomosis

Internal consistency reliability (0.94) was high for ESs; therefore, we used the total mGEARS ratings for further analysis. The inter-rater reliability (0.61) and test–retest reliability (0.64) were moderate. A total score was calculated of the combined performance ratings of the three part procedures.

ESs could discriminate between novice surgeons and experienced surgeons (*p* = 0.02). ES were not able to discriminate between novice surgeons and intermediate surgeons (*p* = 0.79) or intermediate surgeons and experienced surgeons (*p* = 0.07).

No specific type of CW seemed to correlate better with the rating of ESs than others based on age or gender. We found a significant difference between types of CW according to health education background (*p* = 0.047) with a better correlation to ESs for the CW without a health education background (Table 2).

**Table 2:** Demographics of the CWs and their answers to the final questionnaire. The independent *t* test is calculated between the mean delta scores of the CWs

	CWs	Mean delta score (SD)	*p* value
**Demographics**			
Total, *n*	149		
Age, median (range)	65 (19–84)	− 0.025	
Sex			
Woman, *n* (%)	74 (49.7%)	6.00	
Man, *n* (%)	75 (50.3%)	5.52	
Healthcare education			
No,* n* (%)	124 (83.2%)	5.65	
Yes, *n* (%)	25 (16.8%)	6.29	
Social and health care worker, *n* (%)	12 (8.1%)		
Nurse, *n* (%)	8 (5.4%)		
Physiotherapist, *n* (%)	3 (2%)		
Medical secretary, *n* (%)	1 (0.7%)		
**Final questionnaire**^a^			
In the future, would you be interested in helping evaluate surgical videos?			
Yes, *n* (%)	123 (84.8%)	5.93 (1.47)	0.01*
No, *n* (%)	12 (8.3%)	4.73 (1.10)	
Unknown, *n* (%)	10 (6.9%)		
Would you be interested in evaluating videos based on real surgical videos if the identity of the patient was anonymous?			
Yes, *n* (%)	124 (85.5%)	5.86 (1.47)	0.09*
No, *n* (%)	12 (8.3%)	5.10 (1.17)	
Unknown, *n* (%)	9 (6.2%)		
How many hours a month would you be willing to use on video rating?			
Median in h (range)	3.0 (0–30)	5.76 (1.47)	0.56
Would you be willing to evaluate surgical videos without payment to help the education of surgeons?			
Yes, *n* (%)	114 (78.6%)	5.84 (1.47)	0.15
No, *n* (%)	11 (7.6%)	5.19 (1.08)	
Unknown, *n* (%)	20 (13.8%)		
If you were to get paid, how much would you like per video?			
Median in $ (range)	3.8 (0–76.2)	5.76 (1.47)	0.94

*CW* crowd workers

*p < 0.05

^a^Based on 145 CWs

According to the questions on the final questionnaire, there was no correlation between the CWs’ opinion about a future role in the crowd-sourced assessment and the ratings of the CWs, except for the CWs who answered ‘No’ to contribute to the assessment in the future (Table 2).

The Chi-square test showed no significant differences between the skill level of the surgeons and the CWs’ answers ‘Yes/No’ to the question: ‘Would you trust this doctor to perform robot-assisted surgery on you?’ (Table 3).

**Table 3:** CWs could not discriminate between the experience levels of the surgeons (novice, intermediate, experienced) based on the Chi-square for the question: Would you trust this doctor to perform robot-assisted surgery on you?

	Novices	Intermediates	Experienced	Total, *n*
Yes*, n*	248	259	258	765
No*, n*	255	235	235	725
Total,* n*	503	494	493	1490

## Discussion

We found no agreement between the assessments of CWs and ESs when they assessed robot-assisted radical prostatectomies. The CWs were not able to assess the skill levels of the surgeons. This is in contrast to previously published studies, where CWs consistently identified top and bottom performers [2, 10, 17, 18]. These studies only tested the surgeons on basic skills such as laparoscopic peg transfer and not more advanced procedure-specific tasks. Basic surgical skills tasks are simplistic and focus on simple instrument coordination and instrument handling, whereas procedural tasks are complex and include cognitive elements such as planning and complication management [19]. In general, the studies [2, 10, 17, 18] have an unrealistic big difference between novice and experienced surgeons as novice surgeons rarely represent the population to whom the results apply [2, 20]. The novice–expert groups differ too much in skill level and could be too easy to distinguish by both ESs and CWs giving a different result than ours. In contrast, we used where novices were similar to the target group in clinical work. They could be the next to be trained as an experienced surgeon in RARP. Therefore, the groups did not differ as much in skill level compared with former studies, why our CWs had trouble discriminating between the skill level of the surgeons.

Ghani et al. [21] proposed the use of CWs for the assessment of real-life RARPs. With a short introduction to the assessment tool, the CWs were able to identify differences in surgical skill levels. The ESs in their study had no standardized training in video assessment. Rater training aims to improve rater performance by developing the necessary knowledge and skill to reduce rater errors [22]. We rater trained the ESs prior to training to secure the ESs as a ‘gold standard’. We chose not to rater train the CWs to resemble the expected use of crowd-sourced assessment in the future. CWs are laypersons and are normally recruited from online platforms, e.g., Amazon Mechanic Turk (AMT) and C-SATS [2]. They do not receive specific training in surgical skills or how to perceive a good surgeon. The idea is that the CWs have the power in numbers, and therefore can produce results similar to those of experienced surgeons. The span between CWs and ESs in our study is bigger and more realistic than Ghani et al. and could explain the big difference between the ratings of CWs and ESs we found.

We investigated if the CWs could differentiate skill levels based on total performance and not just the mGEARS score. The CWs could not discriminate between the experience level of the surgeons (novices, intermediates, experienced) based on the question ‘Would you trust this doctor to perform robot-assisted surgery on you?’. They would happily let novices perform their surgery even though the novices would not be able to perform the surgery in real life. All this could indicate that CWs are not able to assess more complex procedures as real-life surgeries are.

Crowd workers have been suggested as a solution to reduce the reliance on time-consuming and costly video assessments provided by surgical experts [2]. There was a great willingness from the CWs to help with surgical education voluntarily, also from real-life surgeries. We found no correlation between the performance of the CWs and their opinion about a possible future role as CW. A few CWs answered ‘No´ to this question and they had a score closer to the experienced raters than the other CWs. The scores differ greatly from the scores of the experienced raters and might not have clinical relevance. There was no difference in sex, age, or healthcare education between the groups. They all expressed that they found it difficult and had doubts whether their ratings were good enough. These comments were found among all CWs. We can only speculate, why this small group of CWs performed better and it needs to be assessed in further research.

We were limited by a low number of videos for assessment. The low inter-rater reliability for the ESs (*α* = 0.61) suggests a moderate correlation between the two experienced surgeons. The second rater tended to rate the videos several points lower than the first rater. This could have become an issue if we were to set a pass/fail level, where we could risk passing or failing the wrong surgeons [23]. Higher inter-rater reliability between the experienced surgeons could perhaps be obtained with the addition of more experienced surgeons as raters. Only two experienced RARP surgeons were chosen as video rating is resource intensive and two raters have previously been shown to be sufficient for reliable expert ratings [4, 24, 25]. Further, the ESs only had the first 5 min of the videos to rate, and this could have given them a harder time giving an accurate rating even though they had received rater training before. This is not in accordance with a previous study, where ESs showed agreement for videos edited to the first part of the procedure [14]. The use of videos from a simulated environment instead of real-life surgeries made it possible to completely standardize the procedures and allowed all participants to perform in an independent and unsupervised (“real”) fashion. It is unknown how well the CWs performed on each domain of the assessment tool. We do not know if they understood the domains or if they were able to assess the surgical skills correctly. Future studies using more complex procedures instead of simple standardized tasks are still necessary before abandoning the idea of using laypersons to provide feedback on advanced surgical procedures. More research is needed to identify which types of surgical procedures and assessment tools are suitable for crowd-sourced assessment and how to further standardise the video assessment of CWs [2] as our study suggests that crowd-sourced assessment might not always be useful for assessing surgical skills. Therefore, it is still important for surgeons to focus on other types of feedbacks such as simulation-based testing, and one-to-one instructions by expert surgeons whilst we wait for other innovative assessment methods to be developed such as artificial intelligence.

## Conclusion

We found no agreement between the ratings of CWs and ESs when they assessed robot-assisted radical prostatectomies. As opposed to ESs, CWs could not discriminate between surgical experience using the mGEARS ratings or when asked if they wanted the surgeons to perform their robotic surgery. We still need to investigate whether this method can be used to reduce the dependency on experienced surgeons during surgical training programs before implementing it in clinical everyday life.

## Data Availability

Data available on request from the authors.

## Abbreviations

RARP: Robot-assisted radical prostatectomy
CWs: Crowd workers
ESs: Experienced RARP surgeons
mGEARS: Modified global evaluative assessment of robotic surgery
ANOVA: An analysis of variance
BND: Bladder neck dissection
NVBD: Neurovascular bundle dissection
UVA: Urethrovesical anastomosis
